# Pediatric MDS and bone marrow failure-associated germline mutations in *SAMD9* and *SAMD9L* impair multiple pathways in primary hematopoietic cells

**DOI:** 10.1038/s41375-021-01212-6

**Published:** 2021-03-17

**Authors:** Melvin E. Thomas, Sherif Abdelhamed, Ryan Hiltenbrand, Jason R. Schwartz, Sadie Miki Sakurada, Michael Walsh, Guangchun Song, Jing Ma, Shondra M. Pruett-Miller, Jeffery M. Klco

**Affiliations:** 1 Department of Pathology, St. Jude Children’s Research Hospital, Memphis, TN USA; 2 Department of Pediatrics, Vanderbilt University Medical Center, Nashville, TN USA; 3 Center for Advanced Genome Engineering, St. Jude Children’s Research Hospital, Memphis, TN USA

**Keywords:** Cancer models, Cell signalling

## Abstract

Pediatric myelodysplastic syndromes (MDS) are a heterogeneous disease group associated with impaired hematopoiesis, bone marrow hypocellularity, and frequently have deletions involving chromosome 7 (monosomy 7). We and others recently identified heterozygous germline mutations in *SAMD9* and *SAMD9L* in children with monosomy 7 and MDS. We previously demonstrated an antiproliferative effect of these gene products in non-hematopoietic cells, which was exacerbated by their patient-associated mutations. Here, we used a lentiviral overexpression approach to assess the functional impact and underlying cellular processes of wild-type and mutant *SAMD9* or *SAMD9L* in primary mouse or human hematopoietic stem and progenitor cells (HSPC). Using a combination of protein interactome analyses, transcriptional profiling, and functional validation, we show that SAMD9 and SAMD9L are multifunctional proteins that cause profound alterations in cell cycle, cell proliferation, and protein translation in HSPCs. Importantly, our molecular and functional studies also demonstrated that expression of these genes and their mutations leads to a cellular environment that promotes DNA damage repair defects and ultimately apoptosis in hematopoietic cells. This study provides novel functional insights into SAMD9 and SAMD9L and how their mutations can potentially alter hematopoietic function and lead to bone marrow hypocellularity, a hallmark of pediatric MDS.

## Introduction

Pediatric myelodysplastic syndromes (MDS) are bone marrow (BM) neoplasms that are characterized by hematopoietic cell dysfunction, an increased risk of developing acute myeloid leukemia (AML), and a poor prognosis [1]. Unlike MDS in adults, children with MDS more commonly have BM hypocellularity, a higher frequency of chromosome 7 deletions (monosomy 7), and a distinct set of genetic alterations, as previously shown by our group [2]. We recently reported on the genomic landscape of pediatric primary MDS and identified germline mutations in sterile alpha motif (SAM) domain-9 (*SAMD9*) and its paralog, SAMD9-like (*SAMD9L*), in 17% of pediatric MDS patients [2], and similar findings have been reported by others [2, 3]. We further demonstrated that several germline mutations in SAMD9 (SAMD9-E1136Q) or SAMD9L (SAMD9L-H880Q, -W1180R, and -R1281K) caused significant decreases in proliferation and cell cycle progression in non-hematopoietic cells [3–5]. *SAMD9* and *SAMD9L* are inflammatory-inducible genes located at human 7q21 and, surprisingly, these germline mutant alleles are preferentially absent in the cells with monosomy 7, a process known as adaptation by aneuploidy, suggesting a strong selective pressure against expression of these mutations in hematopoietic cells [3, 4]. Similar mutations in *SAMD9* and *SAMD9L* have previously been observed in disorders associated with BM abnormalities, such as MIRAGE syndrome and Ataxia-Pancytopenia syndrome (ATXPC), respectively [6–9]. Although the loss of *Samd9l* in mice results in a myelodysplasia-like phenotype [10], the function of SAMD9 and SAMD9L, and the impact of pathogenic germline mutations, in hematopoietic cells has yet to be determined.

SAMD9 and SAMD9L are large proteins (>1500 amino acids) and share ~60% amino acid identity, with conserved functional domains, including those involved in DNA/RNA-binding, protein binding, apoptosome formation, and NTP hydrolysis activity [11]. This complex structural feature suggests that the expression of these genes could lead to multiple phenotypes depending on the cellular environment and context. In support of this hypothesis, SAMD9 and SAMD9L are known Poxvirus restriction factors and are inhibited by directly binding to virally encoded proteins [12, 13]. Further, *Samd9l* knockout mice have defects in endosomal processing and fibroblasts from SAMD9-mutant patients have variable defects in Rab5-positive early endosome size [12–14]. In contrast, the mutations associated with MDS, MIRAGE, and ATXPC lead to growth arrest. Importantly, the majority of the described pathogenic germline mutations in SAMD9 and SAMD9L occur in the C-terminal half of the protein within the highly conserved APAF-1-like domain, which contains the predicted core functional NTPase domain [3–5, 7, 13]. This suggests that this domain is critical for SAMD9 and SAMD9L function in hematopoietic cells and in the development of pediatric MDS.

In this study, we used an ex vivo overexpression model to decipher the cellular function of SAMD9 and SAMD9L proteins in primary human and mouse hematopoietic cells. Using a range of proteomics, transcriptomics, and cellular assays, we provide evidence that SAMD9 and SAMD9L regulate multiple key cellular processes, which can lead to cellular stress when dysregulated. We show that overexpression of SAMD9 and SAMD9L regulates hematopoietic cell proliferation, cell cycle, protein translation, DNA damage response, and apoptosis. Importantly, the pathogenic mutations in either SAMD9 or SAMD9L intensify these phenotypes. Revealing the functionality of SAMD9 and SAMD9L, as well as the impact of their pathogenic mutations, is a critical first step in understanding the development of pediatric MDS and potentially other pediatric hematopoietic disorders characterized by BM hypocellularity, such as BM failure syndromes.

## Results

### SAMD9 and SAMD9L regulate hematopoietic cell proliferation and differentiation

**Fig. 1:**
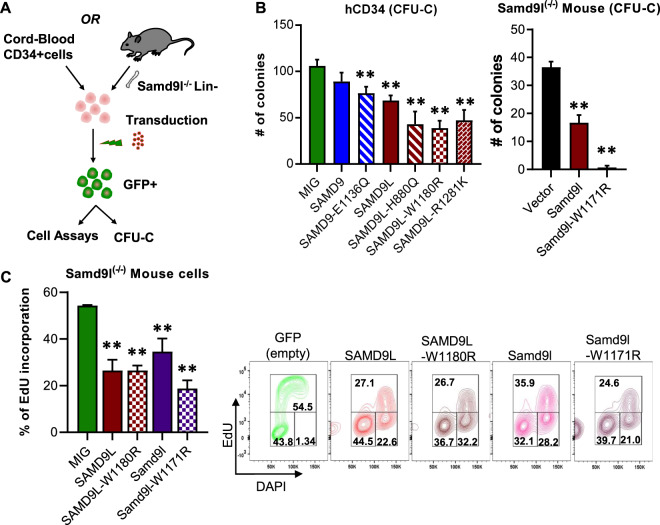
SAMD9 and SAMD9L alter hematopoietic cell proliferation, differentiation, and cell cycle. **A** The experimental model of lentivirus-mediated overexpression of SAMD9, SAMD9L, and their mutations SAMD9-E1136Q, SAMD9L-H880Q, -W1180R, and -R1281K, in cord blood human CD34+ or Samd9l and Samd9l-W1171R in HSPCs cells from Samd9l^−/−^ mice. **B** Colony forming units (CFU-C) showing the total number of colonies from CD34+ (*n* = 4) (left) and Samd9l^−/−^ HSPCs (*n* = 3) (right) transduced with GFP vector control (MIG), SAMD9, SAMD9L, or the indicated pathogenic mutations. Statistics: one-way ANOVA with Bonferroni-correction (ns not significant; **p < 0.01). Error bars indicate standard error of the mean for three biological replicates compared to GFP vector control. **C** Flow cytometric analysis showing the percentage of EdU incorporation in Samd9l^−/−^ HSPCs (*n* = 3) transduced with SAMD9L or Samd9l or their mutations. Statistics: one-way ANOVA with Bonferroni-correction (ns not significant, *p < 0.05, **p < 0.01). Error bars indicate standard error of the mean from three or more biological replicates compared to GFP vector control.

Our previous studies in HEK293T cells demonstrated that exogenous expression of SAMD9 or SAMD9L resulted in reduced cell growth and that this effect was exacerbated by patient-specific mutations [3–5]. We developed a lentiviral overexpression model of SAMD9 and SAMD9L in primary human or mouse hematopoietic stem and progenitor cells (HSPC) to test the effects of these mutations in a more relevant hematopoietic system (Fig. 1A). This approach uses a lentiviral MSCV-IRES-eGFP (MIG) vector to overexpress wild-type SAMD9, SAMD9L, or mutations that we have previously identified in children with MDS (SAMD9: E1136Q; SAMD9L: H880Q, W1180R, or R1281K) [3, 4]. While human cells express both *SAMD9* and *SAMD9L*, the mouse genome only encodes for *Samd9l*, likely due to evolutionary conservation and functional redundancy with *SAMD9* [14, 15]. By using lineage-depleted (Lin^−^) HSPC from a previously characterized *Samd9l*^−/−^ mouse model [10], we are able to validate our studies in a *Samd9l*-null background that both eliminates basal expression and allows us to determine the functional and homologous redundancies between human SAMD9L, human SAMD9, and mouse Samd9l.

Overexpression of wild-type or mutant SAMD9 and SAMD9L in human CD34+ cells leads to a clear suppression in colony formation (Fig. 1B). Subtyping of the colonies shows a variable enrichment of CFU-GM colonies in the SAMD9 and SAMD9L samples relative to the GFP vector control, accompanied by a loss in BFU-Es and the less-committed multipotential colonies, CFU-GEMM (Fig. S1A). This is also further confirmed by flow cytometry studies, where we observed a proportional increase in CD45+CD11b+ cells over CD45−CD71+ cells in all the SAMD9 and SAMD9L groups compared to the control (Fig. S1B). Similarly, overexpression of wild-type mouse Samd9l in *Samd9l*^−/−^ murine HSPCs showed a significant reduction in the number of CFU-Cs relative to the control, and this effect was markedly enhanced by expressing a W1171R mutation (similar to human W1180R) (Figs. 1B and S1C). These changes in CFU numbers were further supported by alterations in cell cycle, namely an accumulation in G2/M phase and decreases in S-phase in both CD34+ cells and Samd9l^−/−^ HSPCs (Fig. S1D) compared to vector controls. These data were confirmed by assessing DNA synthesis at S-phase using the EdU incorporation assay. Overexpression of human or mouse SAMD9L and their homologous W1180R/W1171R mutations lead to a significant decrease in cells in S-phase and an accumulation in G2/M, and the mouse Samd9l mutation intensifies this effect (Fig. 1C). Together our data suggest that overexpression of SAMD9 or SAMD9L impairs hematopoietic cell growth and that the expression of their pathogenic mutations augments this phenotype.

### SAMD9 and SAMD9L protein interactome and the regulation of several pathways including ribosome assembly

**Fig. 2:**
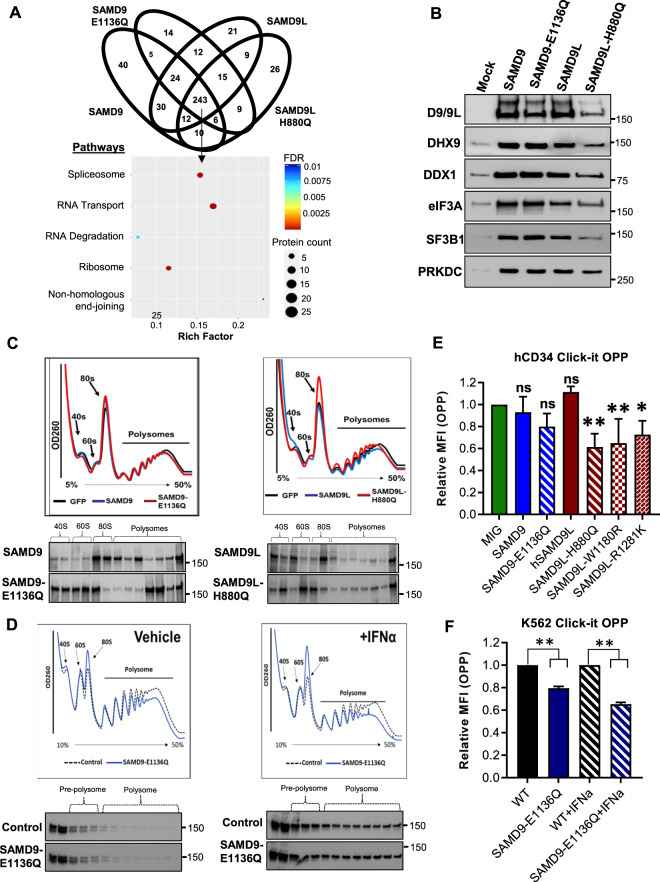
Proteomics reveal a role in RNA processing and protein synthesis. **A** Venn diagram and rich factor graph showing KEGG pathway analysis of shared interacting proteins between wild-type and mutant SAMD9 and SAMD9L. Proteins selected for analysis have a *p* value <0.05 and a control-to-bait ratio >5.0. Rich factor is calculated by statistically significant proteins divided by total proteins, the size of each dot represents protein count, the color of each dot represents FDR significance for the indicated pathway. **B** Immunoprecipitation and western blot analysis for wild-type and mutant SAMD9 and SAMD9L of select proteins from the most enriched KEGG pathways in HEK293T cells. **C** Polysome profile from sucrose gradients (top) of HEK293T cells transfected with SAMD9, SAMD9-E1136Q, SAMD9L, or SAMD9L-H880Q for 16 h. Western analysis (bottom) of SAMD9, SAMD9L, or their variants from sucrose gradient fractions isolated by chloroform/methanol extraction. **D** Polysome profile from sucrose gradients (top) of WT and engineered K562 cell lines containing SAMD9-E1136Q treated with IFNα for 24 h. Western analysis (bottom) of SAMD9 from sucrose gradient fractions isolated by chloroform/methanol extraction. **E** Flow cytometric analysis of protein synthesis rates (OPP incorporation) showing relative mean fluorescence intensity (MFI) of CD34+ cells (*n* = 3) 48 h after transduction with the indicated genes. **F** Flow cytometric analysis of protein synthesis rates in WT and engineered K562 cell lines (*n* = 6) containing SAMD9-E1136Q treated with IFNα for 24 h. One-way ANOVA with Bonferroni-correction (ns not significant, *p < 0.05, **p < 0.01). Error bars indicate standard error of the mean for three biological replicates.

We next took a proteomics approach to understand the function of the SAMD9 and SAMD9L proteins. We used APEX2, a proximity-induced labeling system that is able to effectively mark proteins within a 20 Å radius with biotin to capture both dynamic and stable protein interacting partners [16]. HEK293T cells were co-transfected with a GFP-SAMD9 or GFP-SAMD9L fusion (including mutations) and a GFP Binding-Protein APEX2 fusion (GBP-APEX2). Biotin labeled protein partners were isolated from cells using streptavidin resin and analyzed using mass spectrometry or western blotting. This approach revealed a significant number of interacting proteins (*p* value <0.05), many of which were present in all conditions compared to vector control. SAMD9 and SAMD9-E1136Q shared 66.2% interacting proteins, while SAMD9L and SAMD9L-H880Q shared 66.9%. SAMD9 and SAMD9L shared 72.4% of interacting proteins, indicating significant redundancy in the interactome of SAMD9 and SAMD9L. There were 243 interacting proteins with a ≥5-fold increase in abundance shared amongst all four genotypes relative to vector controls (Fig. 2A). String-based KEGG pathway analysis [17] of the 243 shared proteins revealed several enriched pathways, including ribosome, spliceosome, RNA transport, and DNA repair (Figs. 2A and S2A). Interactions with key proteins from these pathways (DHX9, DDX1 eIF3A, PRDKC, and SF3B1) were validated by IP-western blot (Fig. 2B).

### SAMD9 and SAMD9L play a role in ribosome assembly and consequently in protein synthesis

Given the enrichment of ribosomal proteins (15 of the 243 common proteins) in the SAMD9/SAMD9L interactome, we sought to determine if SAMD9 and SAMD9L associate with different ribosomal subunits during polysome formation. We performed polysome profiling in HEK293T cells and isolated proteins from sucrose gradient fractions using methanol:chloroform extractions as previously described [18]. Overexpression of SAMD9 and SAMD9L and their mutations altered ribosome assembly distribution with an accumulation in the 80S peak (Fig. 2C). Western blot analysis of sucrose gradient fractions confirmed this observation and showed a clear association of SAMD9 and SAMD9L with the ribosomal assembly components by their co-elution with positive controls RPS6 and RPL11 [18] and the absence of the negative control, αTubulin (Figs. 2C and S2B). The majority of SAMD9 associates with pre-polysome (subunits and monosomes) and early polysome complexes while the SAMD9-E1136Q mutation had a strong association with pre-polysomes and late polysomes. Both SAMD9L and SAMD9L-H880Q have a majority pre-polysome assembly pattern.

To further validate this phenotype, we used CRISPR-Cas9 engineering to establish a K562 cell line with a SAMD9-E1136Q mutation. Notably, K562 cells are tetraploid for chromosome 7 and we modified two of the four alleles, thus mimicking the heterozygous mutation state observed in patients while maintaining its physiological expression levels through its endogenous locus. We then performed polysome profiling assays with IFNα treatment to induce SAMD9 expression. These modified isogenic K562 cells have perturbations in their polysome profile, when compared to control K562 cells, which are similar to those observed in our overexpression models, and IFNα treated cells exacerbate this phenotype (Figs. 2D and S2C).

The association of SAMD9 and SAMD9L with proteins in the ribosome and translation pathways suggests that protein synthesis may be altered by SAMD9 or SAMD9L expression. Using the O-propargyl-puromycin (OPP) Click-it assay, which quantifies the incorporation of a puromycin analog into newly synthesized peptides [19], we found that both wild-type SAMD9 and SAMD9L suppressed protein synthesis rate and that the pathogenic mutants exacerbated this phenotype in HEK293T cells (Fig. S3A). Notably, overexpression of wild-type SAMD9, SAMD9L, or Samd9l did not affect protein synthesis in CD34+ and Samd9l^−/−^ HSPCs, whereas the pathogenic mutants suppressed protein synthesis (Figs. 2E and S3B–D), clearly demonstrating a mutant specific phenotype in primary hematopoietic cells. Similarly, CRISPR-engineered K562 cells expressing SAMD9-E1136Q show a decrease in translation compared to control cells, which is further exaggerated in IFNα treated cells compared to the control (Fig. 2F). Together, these data show that SAMD9 and SAMD9L interact with ribosomal assembly complexes and their pathogenic mutations lead to suppression of protein translation in hematopoietic cells.

### Functional domains of SAMD9 and SAMD9L influence their cellular phenotypes

**Fig. 3:**
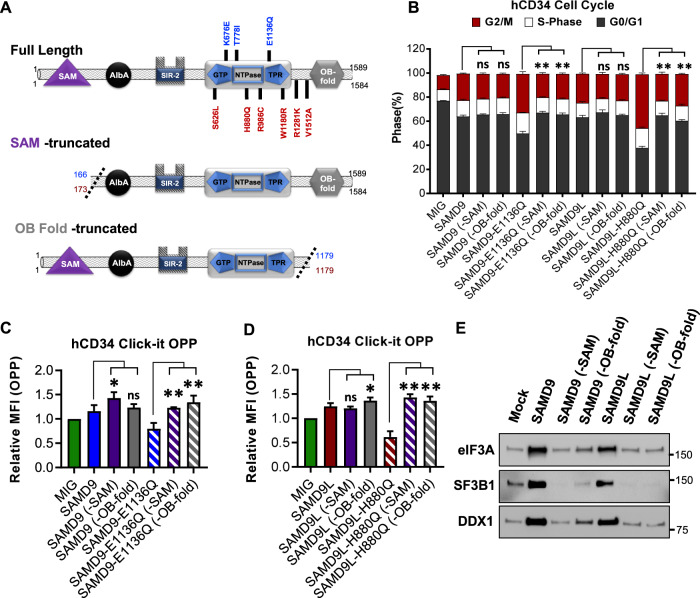
Functional dependency of SAMD9 and SAMD9L on the SAM and OB-fold domains. **A** Top, illustrative representation of full-length proteins of SAMD9 and SAMD9L showing the mutations previously reported by our group in blue and red [3, 4], respectively. SAM- and OB-fold functional domain truncations of SAMD9 and SAMD9L are shown in the middle and bottom, respectively [4, 5]. **B** Flow cytometric analysis demonstrating the cell cycle phases of CD34+ cells (*n* = 3) 48 h after transduction with indicated SAMD9 and SAMD9L truncations. DNA content was stained by NuclearMask. One-way ANOVA with Bonferroni-correction (ns not significant, *p < 0.05, **p < 0.01) for G2/M. Error bars indicate standard error of the mean from three biological replicates compared to full-length controls. Flow cytometric analysis of OPP incorporation showing relative MFI of CD34+ cells (*n* = 3) 48 h after transduction with (**C**) SAMD9 truncations, or (**D**) SAMD9L truncations. **E** Immunoprecipitation and western blot analysis in HEK293T cells of wild-type and the indicated domain deletions of SAMD9 or SAMD9L for top interacting proteins. Statistics: one-way ANOVA with Bonferroni-correction (ns not significant, *p < 0.05, **p < 0.01.

We next sought to assess how the conserved domains within SAMD9 and SAMD9L impacted these cellular phenotypes using a series of domain-based truncations with or without patient-derived mutations (Figs. 3A and S4A, B). Interestingly, the deletion of either the amino-terminal SAM domain (protein/RNA interaction domain) or the carboxy-terminal OB-fold domain (DNA/RNA-binding domain) of SAMD9 or SAMD9L negated the mutation-dependent cell cycle phenotype in CD34+ cells (Fig. 3B). Consistently, we observed a similar pattern of protein synthesis rescue upon expression of each truncation in both human CD34+ cells and Samd9l^−/−^ HSPCs (Fig. 3C, D and S4C). Collectively, these data suggest that the SAM and OB-fold domains are critical functional domains required for cell cycle and translation regulation. To address whether these phenotypes could be associated with changes in subcellular localization resulting from the pathogenic mutations or domain truncations, we next evaluated their expression patterns by confocal microscopy. Overexpression of SAMD9, SAMD9L, and their variants showed distinct patterns of localization confined to the cytosol (Fig. S4D), as previously described [15]. Despite reports that SAMD9 and SAMD9L regulate endosome fusion, colocalization with RAB5, a marker of early endosomes, was not observed [10]. Noticeably, while SAMD9 and SAMD9-E1136Q expression leads to a punctate pattern, SAMD9L and SAMD9L-H880Q expression is exclusively diffuse throughout the cytosol. The deletion of the SAM domain of SAMD9 completely abolished puncta formation, unlike the OB-fold domain deletion (Fig. S4D). Consistently, deletion of the SAM domain and the APAF-1-like domain of SAMD9 and SAMD9L completely rescued the cell cycle and protein synthesis phenotypes in HEK293T (Fig. S5A–D). Deletion of the SAM or OB-fold domain alone decreased the interactions with several targets required for protein synthesis initiation, double-strand break repair, and RNA-splicing, including eIF3A, DDX1, and SF3B1, respectively (Fig. 3F). Taken together, these data demonstrate that both the SAM domain and the OB-fold domain are required for SAMD9 and SAMD9L cellular activity.

### Overexpression of SAMD9 and SAMD9L perturbs multiple pathways in CD34+ cells

**Fig. 4:**
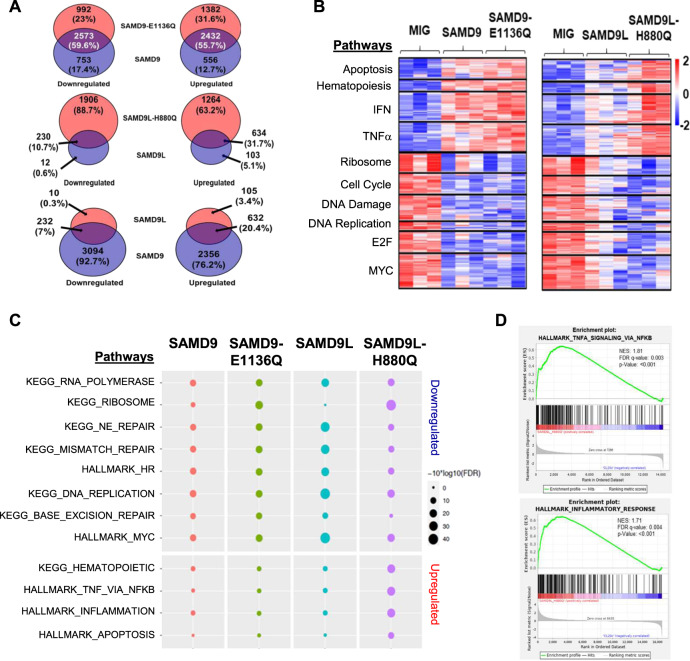
RNA-seq analysis reveals stress response pathways are regulated by SAMD9 SAMD9L expression. **A** Venn diagrams comparing the overlapping of differentially expressed genes in CD34 cells expressing SAMD9, SAMD9-E1136Q, SAMD9L, or SAMD9L-H880Q. **B** Heatmap showing the enrichment of commonly up- and downregulated pathways after wild-type and mutant SAMD9 and SAMD9L expression. The *z*-scale is set to between 2 (red, upregulation) and −2 (blue, downregulation). **C** Rich factor plots of GSEA indicating up- and downregulated pathways in CD34+ cells expressing indicated genes. The size of each dot represents FDR significance for the indicated pathway **D** GSEA illustrating the enrichment of TNFα and inflammatory response target genes in cells expressing SAMD9-H880Q.

We next performed RNA-sequencing in human CD34+ cells overexpressing SAMD9, SAMD9L, or their mutations to further determine the global pathways that are dysregulated by the expression of these genes (Fig. S6A–D). Differentially expressed genes with an FDR ≤0.05 were identified for the different SAMD9 and SAMD9L genotypes relative to control (Fig. 4A). Pathway-enrichment analyses of these differentially expressed genes and gene set enrichment analysis revealed consistent SAMD9- and SAMD9L-dependent upregulation of inflammatory signaling pathways, such as TNFα via NFκB and IFN-α/β, and apoptosis signaling pathways. The downregulated pathways included DNA replication, DNA repair, cell cycle, E2F targets, and MYC pathway targets (Figs. 4B–D and S7A–D and Table S1). Importantly, expression of mutant SAMD9 or SAMD9L leads to further enrichment in these dysregulated pathways. Interestingly, there was consistent downregulation of the minichromosome maintenance complex (*MCM*) family, which has been previously associated with replicative stress in hematopoietic cells, with mutant SAMD9L having the strongest downregulation (Fig. S6E) [19, 20]. Collectively, our transcriptomic analyses indicated a series of cellular stresses and responses to cellular stress (e.g., cell cycle arrest and translation inhibition) mediated by SAMD9 and SAMD9L expression, many of which are exacerbated by pathogenic mutations identified in pediatric MDS.

### SAMD9 and SAMD9L regulate DNA damage repair and apoptosis in hematopoietic cells

**Fig. 5:**
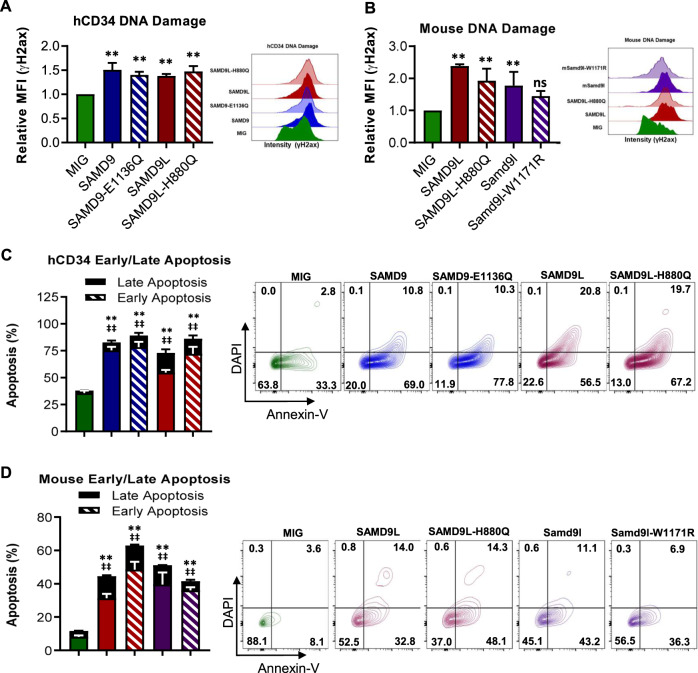
SAMD9 and SAM9L expression induces DNA damage and promotes apoptosis. **A**, **B** Flow cytometric analysis showing the intracellular levels of γH2AX coupled with DNA-staining with NuclearMask. **A**, **B** Histograms and relative MFI of γH2AX in **B** CD34+ (*n* = 3) or **C** Samd9l^−/−^ HSPCs (*n* = 3) transduced with SAMD9, SAMD9L, Samd9l, or their mutations as indicated and cultured for 72 h. One-way ANOVA with Bonferroni-correction (ns not significant, *p < 0.05, **p < 0.01). Error bars indicate standard error of the mean for at least three biological replicates. Flow cytometric analysis demonstrating the percentage of apoptotic cell death measured by annexin V and DAPI 72 h post-transduction. SAMD9, SAMD9L, Samd9l, or the indicated mutations were transduced into **C** CD34+ (*n* = 3) or **D** Samd9l^−/−^ HSPCs (*n* = 3). Early apoptosis was determined as Annexin-V+, DAPI− (striped), and late apoptosis was determined as Annexin-V+, DAPI+ (solid black). One-way ANOVA with Bonferroni-correction (ns not significant, **(early) and ^‡‡^(late) for *p* < 0.01). Error bars indicate standard error of the mean for at least three biological replicates.

Our proteomic studies (Fig. 2A, B) revealed an interaction of SAMD9 and SAMD9L with PRDKC and DDX1, known DNA repair pathway factors [21–23] and our transcriptomic studies also supported a link with DNA damage (Figs. 4B–E and S8A). Therefore, we functionally investigated the link between SAMD9/SAMD9L expression and DNA damage by analyzing gamma-H2AX (γH2AX) levels in CD34+ cells. The expression of SAMD9, SAMD9-E1136Q, SAMD9L, and SAMD9L-H880Q significantly increased the intensity of γH2AX compared to control (Fig. 5A). Similarly, overexpression of human and mouse SAMD9L genes in Samd9l^−/−^ HSPCs also leads to an increase in γH2AX levels (Fig. 5B). Consistent with this DNA repair defect, live cell imaging showed nuclear condensation in *Samd9l*^−/−^ HSPCs overexpressing wild-type or mutant *Samd9l* relative to GFP vector (Fig. S8B).

Our transcriptomics data also pointed to apoptosis as a consequence of SAMD9 and SAMD9L expression (Figs. 4B–E and S8C). Consistent with these transcriptomic data, expression of SAMD9, SAMD9-E1136Q, SAMD9L, and SAMD9L-H880Q caused a significant increase in CD34+ cells undergoing early and late apoptosis compared to the control (Fig. 5C), as measured by Annexin v/DAPI staining. We also observed increased levels of apoptosis in Samd9l^−/−^ HSPCs expressing human SAMD9L, SAMD9L-H880Q, mouse Samd9l, and Samd9l-W1171R (Fig. 5D).

## Discussion

*SAMD9* and *SAMD9L* have recently been described as new germline predisposition genes in pediatric MDS and in several multisystem disorders such as MIRAGE and ATXPC syndromes [3–9]. Many patients with *SAMD9* and *SAMD9L* mutations have outgrowth of cells with non-random loss (or inactivation) of the germline variant, presumably as a cellular adaptation to the mutation-associated growth restriction [2, 4, 5]. The resulting haploinsufficiency of genes located on chromosome 7 can lead to MDS or AML, especially when additional cooperating mutations are somatically acquired [5]. Despite the now strong association of germline *SAMD9* and *SAMD9L* variants with MDS, the cellular impact of both wild-type or mutant SAMD9 (or SAMD9L) in hematopoietic cells has yet to be elucidated. Here, we used a series of proteomic, transcriptomic, and cell biology methodologies to show that mutant SAMD9 or SAMD9L expression results in profound perturbations of cellular processes in hematopoietic cells, including disruption of protein synthesis and cell cycle, while also activating DNA damage responses and apoptosis.

Dysregulation of ribosomal biology and protein synthesis is a common finding in pediatric BM disorders, including Diamond-Blackfan anemia, Shwachman-Diamond syndrome, and dyskeratosis congenital [24–28]. Importantly, these alterations lead to defects in translation and induce ribosomal stress, causing apoptosis [28]. The multi-omic and functional data presented here extend these associations to SAMD9 and SAMD9L-related syndromes. Not only does expression of mutant *SAMD9* or *SAMD9L* impair the rate of protein synthesis, but the SAMD9 and SAMD9L proteins are associated with components of the polysome complex and with proteins linked to ribosome assembly and translation, such as EIF3A and DHX9 [29, 30]. The association of SAMD9 and SAMD9L to protein synthesis defects in hematopoietic cells is perhaps not surprising considering their role as Poxvirus restriction factors, in which they specifically block cap-dependent and -independent translation of intermediate and late viral mRNAs [31]. Despite the relative lack of mutations in components of the splicing machinery in pediatric MDS when compared to MDS in adults [2, 32, 33], the physical proximity of SAMD9 and SAMD9L to RNA helicases and splicing factors, like DDX1 and SF3B1, suggests that RNA processing may be a more conserved feature of MDS across the age spectrum than previously recognized. The RNA-binding domains of SAMD9 and SAMD9L also suggest there may be a direct interaction with RNA transcripts that could regulate both translation and splicing [11]. However, additional mechanistic studies are clearly required to pursue how SAMD9 and SAMD9L influence these processes.

To our knowledge, our data are the first to show that defects in the DNA repair pathway are a functional consequence of SAMD9 and SAMD9L expression. The genomic instability that results from cell cycle arrest, ribosomal perturbations, and DNA damage is a key driver in the development of MDS [34–38]. Alterations of DNA repair genes drive the progression to MDS in Fanconi anemia [39] and unrepaired DNA defects in hematopoietic cells cause remarkable long-term functional perturbations and represent a primary driving force of accrual of additional mutations, which in turn promote clonal expansion and malignant transformation [38, 40–42]. We likewise showed that cells expressing SAMD9 or SAMD9L mutants accumulate in G2/M, a key checkpoint for DNA damage [43, 44]. Consistently, our transcriptomic data further support the potential impact on cell cycle and DNA repair pathways with downregulation of notable genes, such as *MYC*, *CDK6, E2F1*, *POLE*, and several members of the *MCM* family. Intriguingly, all of these genes were further downregulated when pathogenic SAMD9 or SAMD9L mutations were present. Taken together, the observed phenotypes resemble the DNA-replicative stress evoked by *MCM* genes downregulation in aging HSPCs, which is associated with activation of γH2AX alongside cell cycle abnormalities [19, 45].

**Fig. 6:**
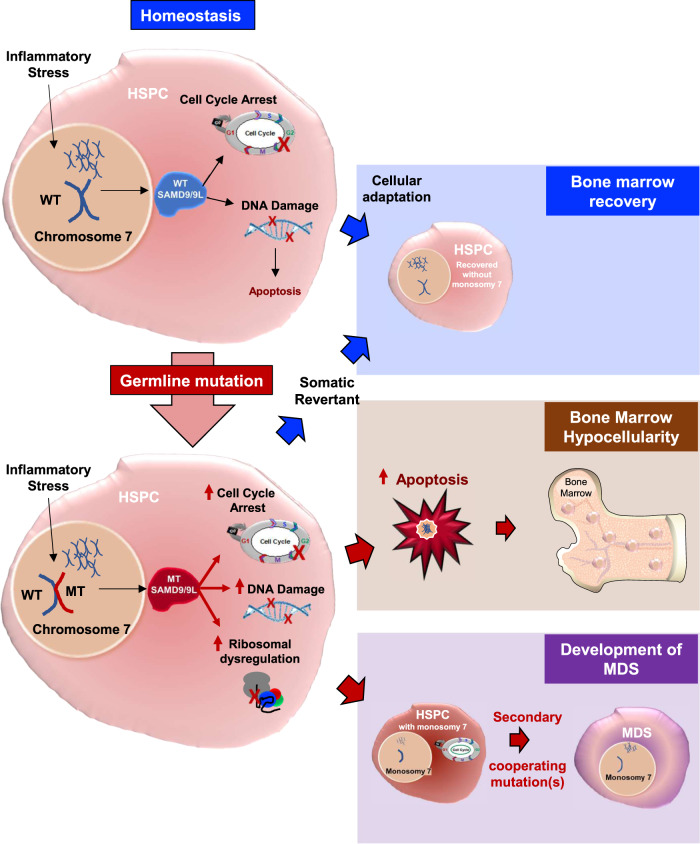
Model of the functional roles of SAMD9 and SAMD9L in hematopoietic cells. SAMD9 and SAMD9L regulate proteins involved in cell cycle, DNA damage repair, and protein synthesis. Mutated proteins (red) can significantly amplify these phenotypes, and if any of these remain unchecked, will ultimately lead to cell death. Collectively these features lead to bone marrow hypocellularity (middle). Alternatively, cells that lack the mutant protein (either by monosomy 7 or somatic reversion can have alternative fates dictated largely by the acquisition of additional somatic mutations. Hematopoietic stem and progenitor cell (HSPC), myelodysplastic syndrome (MDS), wild-type (WT), mutant (MT).

A hallmark of pediatric MDS is BM hypocellularity and low peripheral blood counts [46]. Indeed, many of the SAMD9/SAMD9L mutations originally described by our group in pediatric MDS were found in patients with refractory cytopenia of childhood [2], which is predominantly associated with a hypocellular BM phenotype. Likewise, the study by Bluteau et al. also linked *SAMD9* and *SAMD9L* germline mutations to BM failure syndromes, which are a hypo-proliferative group of BM disorders [7]. This is in stark contrast to the hypercellular BM that are more commonly observed in adults with MDS. These observations alone suggest a distinct pathobiology of MDS in children and adults. As shown in this study, overexpression SAMD9 or SAMD9L in primary hematopoietic cells results in decreased proliferation and increased apoptosis, which would ultimately lead to the hypocellular phenotype observed in patients. We speculate (see Fig. 6) that the observed effects on ribosomal biology, DNA damage, and the resulting genomic instability can drive the observed apoptotic phenotype [26, 30, 40–42], ultimately leading to decrease cellularity in the BM. Unrepaired DNA defects in hematopoietic cells cause remarkable long-term functional perturbations and represent a primary driving force for accrual of additional mutations, which in turn promote clonal expansion and malignant transformation [39, 46–49]. Clinical data suggest that there are multiple adaptive mechanisms to address this cellular stress, including the outgrowth of cells with somatic revertant mutations and chromosome 7 deletions, all of which are potential outcomes of children harboring germline SAMD9 or SAMD9L mutations [3, 6].

Our data also highlight an important functional redundancy between SAMD9 and SAMD9L, including both human and mouse for SAMD9L. Despite their large size (over 1500 amino acids), SAMD9 and SAMD9L share ~60% amino acid identity with multiple conserved sequence domains, and thus their functional redundancy is not surprising. Here, we show that the expression of both proteins leads to largely identical phenotypes in hematopoietic cells, with SAMD9L consistently having the strongest effect. Notably, the human genome encodes for both *SAMD9L* and *SAMD9*, while murine cells only contain *Samd9l*. This lack of redundancy in the mouse genome may explain why the overall phenotypes observed in the *Samd9l-*null cells were more pronounced than those in cord blood CD34 cells. This suggests that murine models may be more advantageous to further map the function of these proteins. Whether these two proteins in humans are truly functionally redundant requires more extensive investigation into their structure and function. One potential source of heterogeneity may be epigenetically based. For example, SAMD9 is considered a constitutive restriction factor for Poxvirus infection, while SAMD9L is induced under inflammatory conditions [13].

In summary, we demonstrated for the first time the immediate impact of SAMD9 and SAMD9L overexpression in primary hematopoietic cells and our work has provided critical insights into the underlying biology that leads to the hypocellular phenotype seen in many children with SAMD9 and SAMD9L germline mutations. Considering that many cases of pediatric MDS, like BM failure syndromes, are defined by BM hypo-proliferation, these findings may extend to a larger percentage of pediatric myeloid disorders and may ultimately provide new therapeutic options for future investigation.

## Methods

### Animals

Samd9l^−/−^ mice were kindly provided by the RIEKN BRC through the National Bio-Resource Project of the MEXT, Japan with approval from Dr. Hiroaki Honda [10]. Animal studies were approved by St. Jude Children’s Research Hospital Institutional Animal Care and Use Committee. Hematopoietic cells were selected from the flushed bones using the lineage-depletion EasySep Mouse HSPC Kit (StemCell Technologies, Canada) [50].

### Cell culture

Human cord blood-derived CD34+ cells (Lonza, Switzerland) were cultured in expansion medium containing StemSpan SFEM-II (StemCell Technologies, Canada) enriched with human cytokines (PeproTech, NJ) including interleukin-6 (100 ng/ml), Fms-like tyrosine kinase-3 ligand (FLT3-L, 100 ng/ml), Stem Cell Factor (SCF, 100 ng/ml), Thrombopoietin (100 ng/ml), 1 µM Stem Regenin-1 and 35 nM UM171 (StemCell Technologies, Canada). For mouse cells, Samd9l^−/−^ HSPCs were expanded overnight in RPMI (ThermoFisher, MA) with 10% FBS and supplemented with the murine (6–8 weeks) cytokines including interleukin-3, interleukin-6, SCF, Thrombopoietin, and FLT3-L (PeproTech, NJ).

### Flow cytometry sorting and analysis

Transduced cells (as measured by GFP positivity) were sorted using the FACSAria sorter (BD Biosciences, CA). Analytical flow cytometry was done using LSR FORTESSAII (BD Biosciences, CA). For intracellular staining, transduced cells were harvested after the indicated time, fixed with 4% paraformaldehyde, permeabilized with 0.5% Triton X-100, and stained with the appropriate antibodies (Table S2). EdU incorporation assay was done using the Click-iT Plus EdU Kit (Invitrogen, CA) after incubating the cells with 10 µM EdU for 2 h as previously described [2]. The translation rates were examined using Click-iT Plus OPP Protein Synthesis Kit (Invitrogen, CA) where the cells were incubated with 10 µM OPP for 30 min and assessed as previously described [50]. DNA damage was measured using anti-phospho-H2AX(Ser139) antibody (BioLegend, CA). For DNA content, cells were stained with NuclearMask (Invitrogen, CA). For apoptosis assessment, transduced cells were cultured for 72 h, blocked with binding buffer with 5% rat serum, and stained with Annexin-V antibody and DAPI [51]. All data were analyzed using FlowJo software (TreeStar, OR) and presented as mean fluorescence intensities or histograms.

### Data presentation and statistical analysis

All graphs were generated using GraphPad Prism 8.0 (San Diego, CA). One-way ANOVA with Bonferroni-correction was used for statistical analyses. Statistical significance was set at *p < 0.05, and **p < 0.01 compared to GFP vector control unless stated differently.

Additional methods are listed in the supplemental data.

## Supplementary information

Supplemental

## Data Availability

RNA-seq data were deposited into Gene Expression Omnibus (GEO) (accession number: GSE152420). IP-MS data were deposited into Proteomics Identification Database (PRIDE) (px-submission #428150).
