# Supplementary material for: Pediatric MDS and bone marrow failure-associated germline mutations in SAMD9 and SAMD9L impair multiple pathways in primary hematopoietic cells
Source: Leukemia. 2021 Mar 17;35(11):3232–44. doi: 10.1038/s41375-021-01212-6 (PMC8446103; doi:10.1038/s41375-021-01212-6)
Supplement: Supplementary file 1 — Supplemental [file 41375_2021_1212_MOESM1_ESM.docx]

**Supplemental data**

**Methods**

***Generation of Mutant K562 cell lines.***

SAMD9-E1136Q K562 cells were generated using CRISPR-Cas9 technology. Briefly, 400,000 K562 cells were transiently co-transfecting with precomplexed ribonuclear proteins (RNPs) consisting of 100 pmol of chemically modified sgRNA (Synthego), 33 pmol of spCas9 protein (St. Jude Protein Production Core), 3ug of ssODN donor (IDT) and 200ng of pMaxGFP (Lonza). The transfection was performed via nucleofection (Lonza, 4D-Nucleofector™ X-unit) using solution P3 and program FF-120 in a small (20ul) cuvette according to the manufacturer’s recommended protocol. Five days post nucleofection, cells were single cell sorted for GFP+ (transfected) cells by FACs and clonally selected. Clones were screened and verified for the desired modifications via targeted deep sequencing using gene specific primers with partial Illumina adapter overhangs as previously described(1). In brief, clonal cell pellets were harvested, lysed and used to generate gene specific amplicons with partial Illumina adapters in PCR#1. Amplicons were indexed in PCR#2 and pooled with other targeted amplicons for other loci to create sequence diversity. Additionally, 10% PhiX Sequencing Control V3 (Illumina) was added to the pooled amplicon library prior to running the sample on an Miseq Sequencer System (Illumina) to generate paired 2 X 250bp reads. Samples were demultiplexed using the index sequences, fastq files were generated, and NGS analysis was performed using CRIS.py(2). Editing construct sequences and screening primers are listed in **Table S3**.

***CFU-C assay.***

Sorted CD34 or Samd9l^-/-^ HSPCs (10,000 cells) from all conditions were cultured in methylcellulose with the appropriate human or mouse cytokines (MethoCult, StemCell-Technologies, Canada) at 37°C with 5% CO2 for 14 or 7 days, respectively. Colonies were then counted using a 4x magnification microscope. For serial-replating assays, methylcellulose was solubilized in PBS, and cells were spun down and replated at 10,000 cells/plate in methylcellulose for another week for two more rounds.

***Lentiviral Production.***

Lentivirus was generated as previously reported(3). Briefly, HEK293T cells were plated at 5x10^6 cells/10ml DMEM substituted with 10%FBS. At ~40% confluency, cells were transfected using the transfection cocktail containing FuGENE HD (Promega, WI), packaging plasmid mixture (pHDM-G, PCAGG-HIVgpco, pCAG4-RTR2), and the plasmid of interest (CL20c-MIG-DEST[SAMD9], [SAMD9 E1136Q], [SAMD9L], [SAMD9L R1281K], [SAMD9L H880Q], [SAMD9L W1180R] [mSamd9l], [mSamd9l-W1171R], or empty vector (CL20c-MIG-DEST). The cells were transfected for 48hrs at 37C. After the transfection period, the virus-containing supernatant was filtered into an Amicon Ultra-15 Centrifugal Filter Unit (Millipore-Sigma, MA) and concentrated at 2000xg.

***Transduction and transfection.***

All plasmids were cloned using the Gateway entry vector (pENTR/D-TOPO) and destination vector pcDNA6.2/N-EmGFP-DEST or CL20c-MIG-DEST as previously described(3) (Lifetech, China). For the truncations, the amino acid positions were selected based on previously described homologous domains and the secondary protein structure prediction tool JPred4(4, 5). HEK293T (400,000 cells) were plated overnight and then transfected with the cloned plasmids using FuGene, as previously described(3). For transduction, K562 cells, hCD34+cells, or Samd9l-/- HSPCs were resuspended in 2X of their appropriate media supplemented with 1ug/ml polybrene (2.5x10^5 cells/well). Cells were transduced with an equal volume of the concentrated lentiviral particles by spinfection at 2000x*g*/90min/30C followed by overnight incubation at 37C.

***Fluorescence Microscopy.***

Transfected HEK293T cells expressing GFP-tagged SAMD9 and SAMD9L constructs were fixed with 4% paraformaldehyde, permeabilized in 0.3% triton X-100, blocked with 5% rat serum, and stained with the indicated primary, secondary antibodies and DAPI (**Table S2**). Coverslips were mounted using ProLong Diamond Antifade (Invitrogen, CA). Images were acquired on a Nikon C2 laser scanning confocal microscope using a 60X oil-objective lens controlled by NIS-Elements software (Nikon, Japan). For live-cell imaging, DNA was stained with DRAQ staining solution (ThermoFisher, MA).

***Mass Spectrometry.***

Immunoprecipitated protein samples were run on a short gel for peptide digestion and extraction as described in a previously published protocol(6). The peptide extracts were loaded on a nanoscale capillary reverse phase C18 column (75 id, 10cm) by a HPLC system (Thermo EASY-nLC 1000). The eluted peptides were ionized by electrospray ionization and detected by an inline mass spectrometer (Thermo LTQ Orbitrap Elite). UniProt human database concatenated with a reversed decoy database for evaluating false discovery rate was used for searching the raw data. Sequest v.28 (rev. 12) search engine was used for database searches(7). The spectral counts between samples for a given protein were used to calculate the p-value which is derived by G-test(8).

***HEK293T Immunoprecipitation***

HEK29T cells were plated and transfected with APEX2-GBP and GFP-tagged constructs and biotinylation, quenching, and cell harvest was done as previously described(9). Cells were lysed in RIPA buffer with protease/phosphatase inhibitor and quantified by Bradford assay. Biotinylated proteins were isolated by incubating lysates (equal total protein concentration) in Streptavidin Magnetic Beads (Pierce, IL) overnight at 4C with rotation. Biotinylated proteins were eluted in 25mM Tris-HCl pH 7.4, 150mM NaCl, 0.5% triton X-100, 5% glycerol, 2% SDS, 10mM BME, and 1mM biotin with boiling.

***HEK293T and K562 Polysome Profiling and Fractionation Assays.***

Polysome profiling and fractionation was done as previously described(10). Briefly, transfected cells were treated with 100ug/mL of cycloheximide for 15min and washed properly. Cells were harvested and lysed in 20mM Tris-HCl pH7.5, 100mM KCl, 5mM MgCl2, protease/phosphatase inhibitor (ThermoFisher, MA) 10U/mL DNAse1, 40U/uL RNasin-PLUS (Promega, WI), 100ug/mL cycloheximide, 0.5% NP-40, 0.5% Deoxycholate) and nucleic acid concentration was quantified at 260nm. 5%-50% sucrose gradients with 40U/uL RNasin-PLUS, 100ug/mL cycloheximide were loaded with equal RNA concentrations. Ultracentrifugation was done using a Beckman ultracentrifuge with a SW60Ti swinging bucket rotor at 27,000 rpm for 174min at 4C with maximum acceleration and brake. Fractions were analyzed and collected using a Gradient Station-ip (BioComp, Canada). Proteins were isolated from each fraction by methanol:chloroform extraction and analyzed by western.

**RNA-Seq read mapping and data analysis**

RNA reads were mapped using our StrongARM pipeline, described previously(11). Paired-end reads from RNA-seq were aligned to the following four database files using BWA: (i) the human GRCh37-lite reference sequence, (ii) RefSeq, (iii) a sequence file representing all possible combinations of non-sequential pairs in RefSeq exons and, (iv) the AceView database flat file downloaded from UCSC representing transcripts constructed from human ESTs. Additionally, they were mapped to the human GRCh37-lite reference sequence using STAR. The mapping results from databases (ii)–(iv) were aligned to human reference genome coordinates. The final BAM file was constructed by selecting the best of the five alignments. Reads from aligned bam files were assigned to genes and counted using HTSeq with the GENCODE human release 19 gene annotation(12). The gene count matrix was generated as well as a FPKM (fragment per kilobase per million) gene expression data matrix using gene length information. Log2 CPM (counts per million) values were used for differential gene expression analysis. We first determined the smallest group with the smallest number of samples among all groups being compared. A cut-off of 10 CPM was used as the threshold to define if a gene is expressed. Genes not meeting this cutoff were excluded from downstream analysis. Batch effect was corrected using the ComBat method available from R package SVA(12). Limma R package was used for differential gene expression analysis. Pathway enrichment/over-representation analysis was done using ClusterProfiler R package(13, 14). Gene Set Enrichment Analysis (GSEA) was done using the Broad Institute GSEA tool(15).

***hCD34+ RNA Extraction and Sequencing***

hCD34+cells were transduced with lentivirus and sorted for GFP+ cells as described above. Total RNA was extracted using quick-RNA Microprep kit (Zymo Research, CA). RNA-sequencing was performed using TruSeq Stranded Total RNA library kit (Illumina, CA) and analyzed, as previously described(3, 11).

**Supplemental Figures**

**
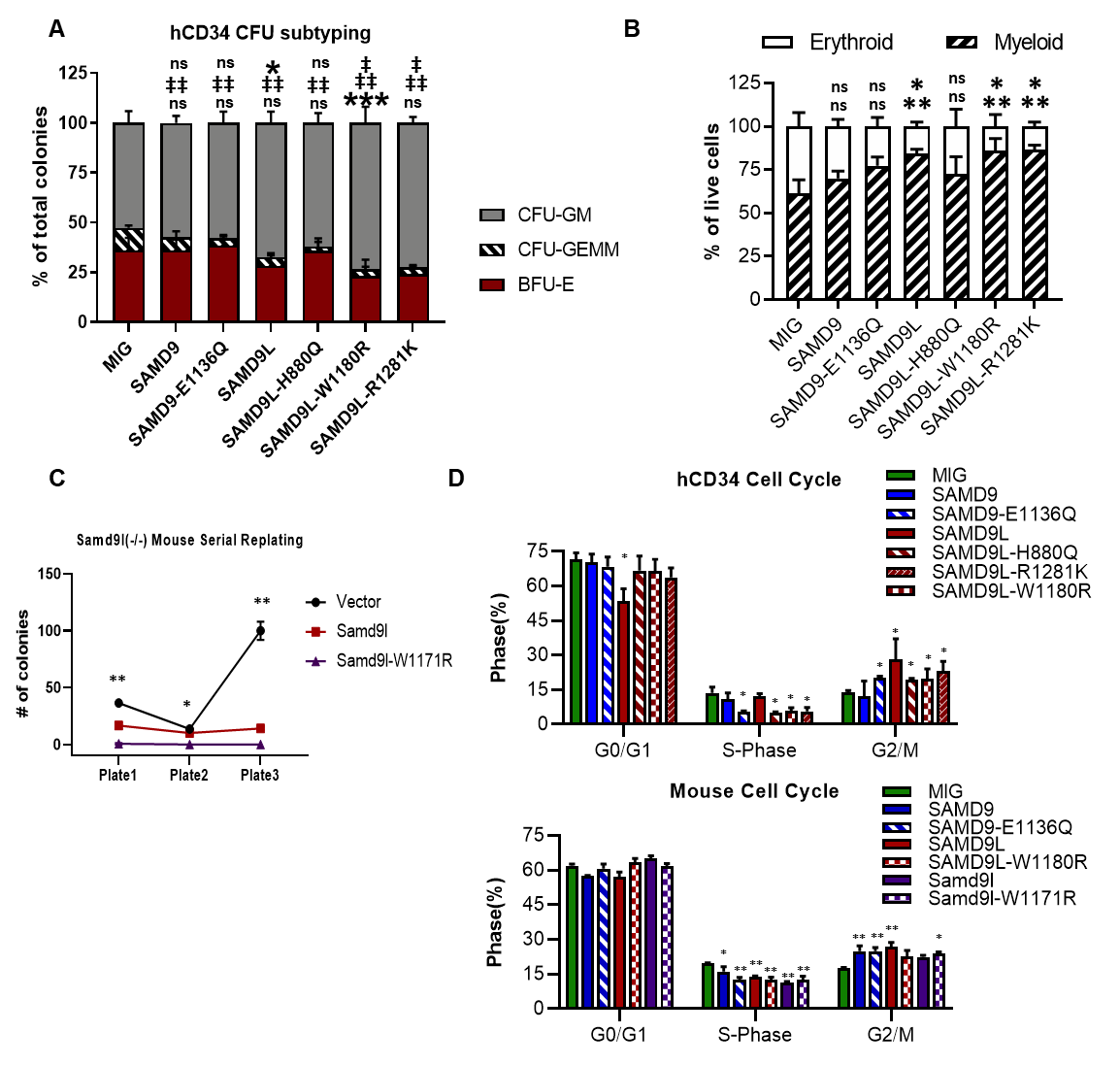
**

**Figure S1. Mutations in SAMD9 and SAMD9L impair hematopoietic proliferation and differentiation** **A**. Subtyping of hCD34+ cells (n=3) by CFU-GM, CFU-GEMM, or BFU-E after lentiviral transduction and sorting for GFP+ cell populations. Statistics: One-way ANOVA with Bonferroni-correction (ns, not significant; *(GM) **(GEMM) ***(E) for p<0.05; ‡(GM) ‡‡(GEMM) ‡‡‡(E) for p<0.01. Error bars indicate standard error of the mean for at least three biological replicates compared to MIG control. **B**. Flow cytometric analysis (n=3) showing the percent of erythroid (CD45-CD71+) or myeloid lineage (CD45+CD11b+) from hCD34+cells 14 days post CFU-C assay. One-way ANOVA with Bonferroni-correction (ns, not significant; *(erythroid) **(myeloid) p<0.01). Error bars indicate standard error of the mean for three biological replicates compared to GFP-vector control. **C**. Serial replating of GFP+ sorted Samd9l-/- HSPCs (n=3) expressing wild-type or mutant Samd9l over 3 weeks. Statistics: ANOVA with Bonferroni-correction (* p<0.05, ** p<0.01). Error bars indicate standard error of the mean for three replicates compared to GFP-vector control. **D**. Flow cytometric analysis of hCD34+ (top) (n=3) or Samd9l^-/-^ HSPCs (n=3) (bottom) showing phases of cell cycle (NuclearMask). Cells were transduced with GFP-vector, *SAMD9*, *SAMD9L,* or their mutations as well as *Samd9l* or its mutation, *Samd9l*-W1171R as indicated.

**
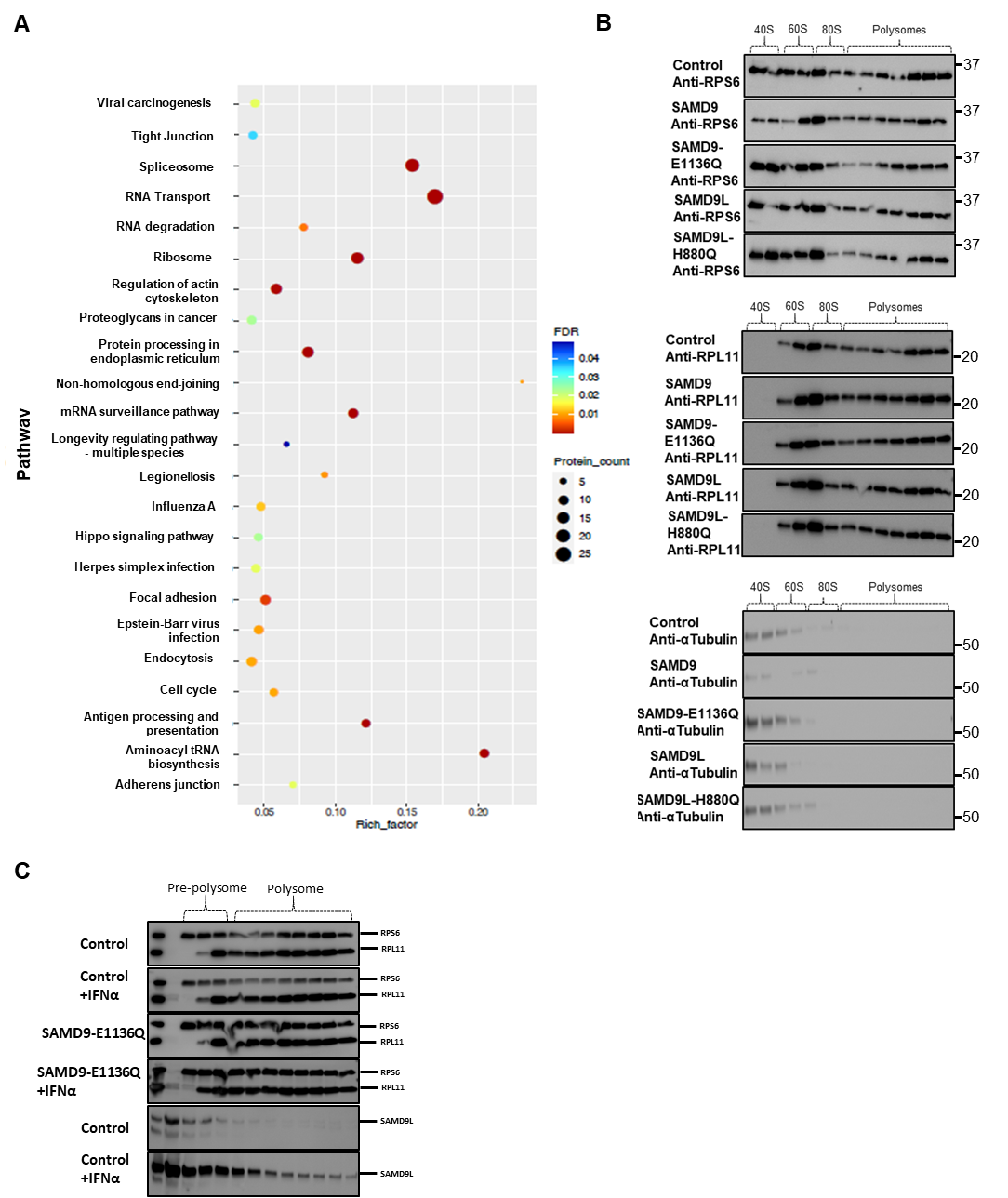
**

**Figure S2. SAMD9 and SAMD9L and their mutations suppress cellular translation.**

**A**. Rich factor graph showing the full list of the KEGG pathway analysis of common interacting proteins between wild-type and mutant SAMD9 and SAMD9L. Selected proteins have a p-value <0.05 and control-to-bait ratio ≤5.0. Rich factor is calculated by statistically significant proteins divided by total proteins, the size of each dot represents protein count, the color of each dot represents FDR significance for the indicated pathway. **B**. Western blot analysis of positive control RPS6 (top) and RPL11 (middle), and negative control αTubulin (bottom) from polysome profile fractions of HEK293T cells expressing wild-type and mutant SAMD9 and SAMD9L. **C**. Western analysis of SAMD9L, RPS6, or RPL11 from sucrose gradient fractions isolated by chloroform/methanol extraction from isogenic or control K562 cells.


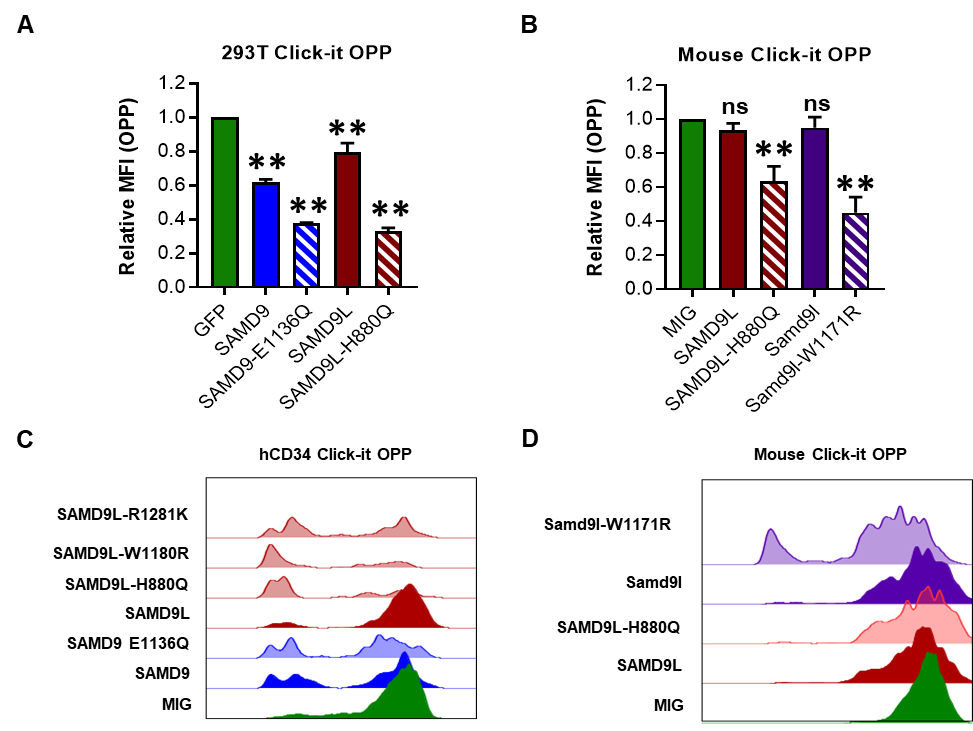


**Figure S3. Translation analysis of overexpressed germline mutations A,B**. Flow cytometric analysis of protein synthesis rates (OPP incorporation) showing relative mean fluorescence intensity (MFI) of HEK293T cells (n=3) (**A**) or Samd9l^-/-^ HSPCs (n=3) (**B**), 48hr after transduction with the indicated genes. One-way ANOVA with Bonferroni-correction (ns, not significant, * p<0.05, ** p<0.01). Error bars indicate standard error of the mean for three biological replicates compared to GFP-vector control. **C,D** Representative histograms of flow cytometric analysis of OPP incorporation assay (total MFI) from GFP+, live cells in (**C**) hCD34+, or (**D**) Samd9l-/- HSPCs 48hr after transduction with the indicated genes.

**
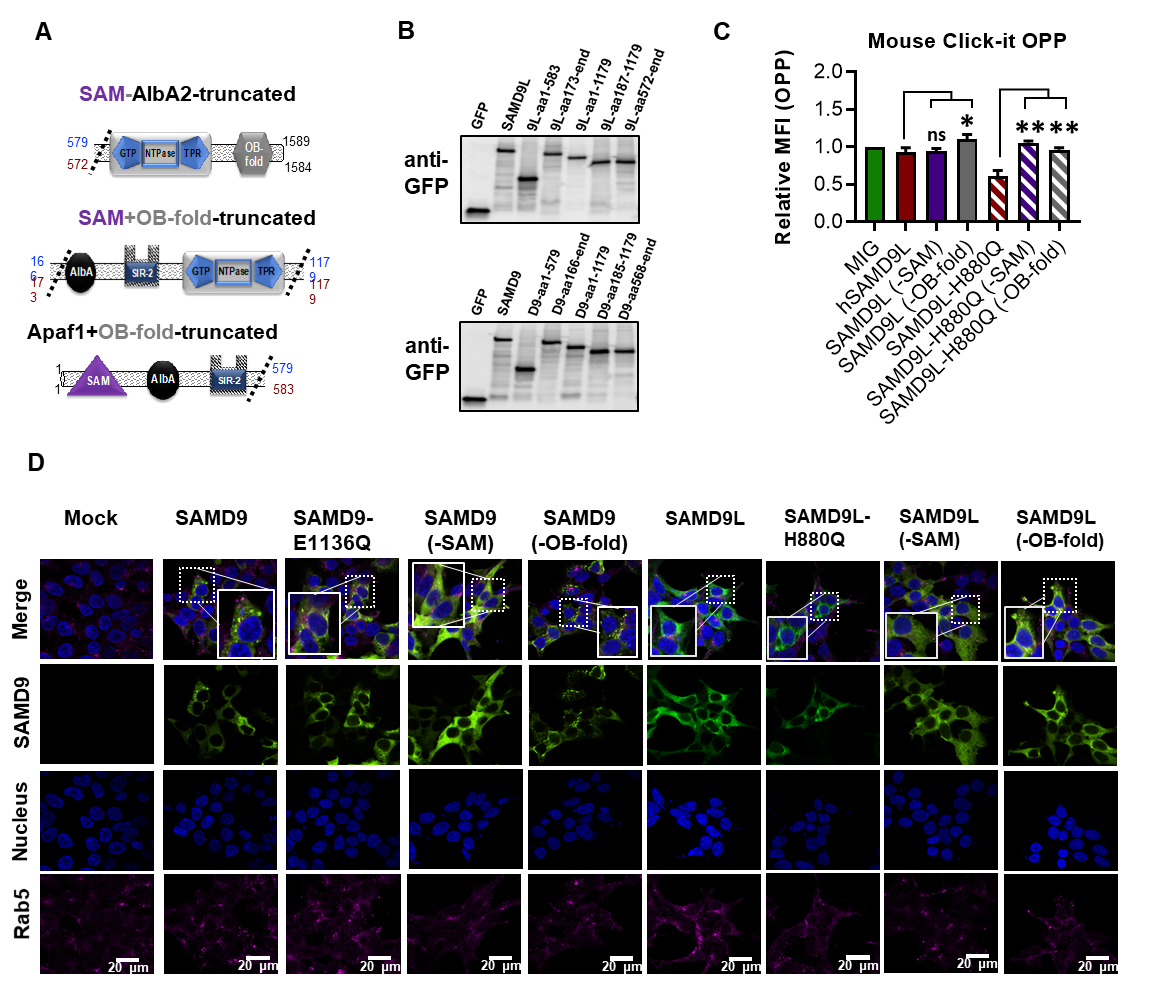
**

**Figure S4. Functional homology-based truncations identified functional-domains in SAMD9 and SAMD9L A**. Illustrative representation of the additional functional-domain-based truncations of SAMD9 and SAMD9L. **B**. Western analysis of SAMD9 and SAMD9L truncations demonstrating stable expression after 16hr in HEK293T cells. **C**. Flow cytometric analysis of OPP incorporation showing relative MFI of Samd9l^-/-^ HSPCs (n=3) expressing SAMD9L truncations. One-way ANOVA with Bonferroni-correction (ns, not significant, * p<0.05, ** p<0.01). Error bars indicate standard error of the mean for three biological replicates compared to GFP-vector control.  **D**. Immunofluorescent confocal microscopy analysis of HEK293T transfected for 16 hours with GFP-tagged SAMD9L truncations as labeled. SAMD9 and SAMD9L are labeled with eGFP (green), Rab5 is labeled with Alexa Fluor 647 (violet), the nucleus is stained with DAPI (blue). Images were acquired on a Nikon C2 laser scanning confocal microscope using a 60X oil immersion optical lens.


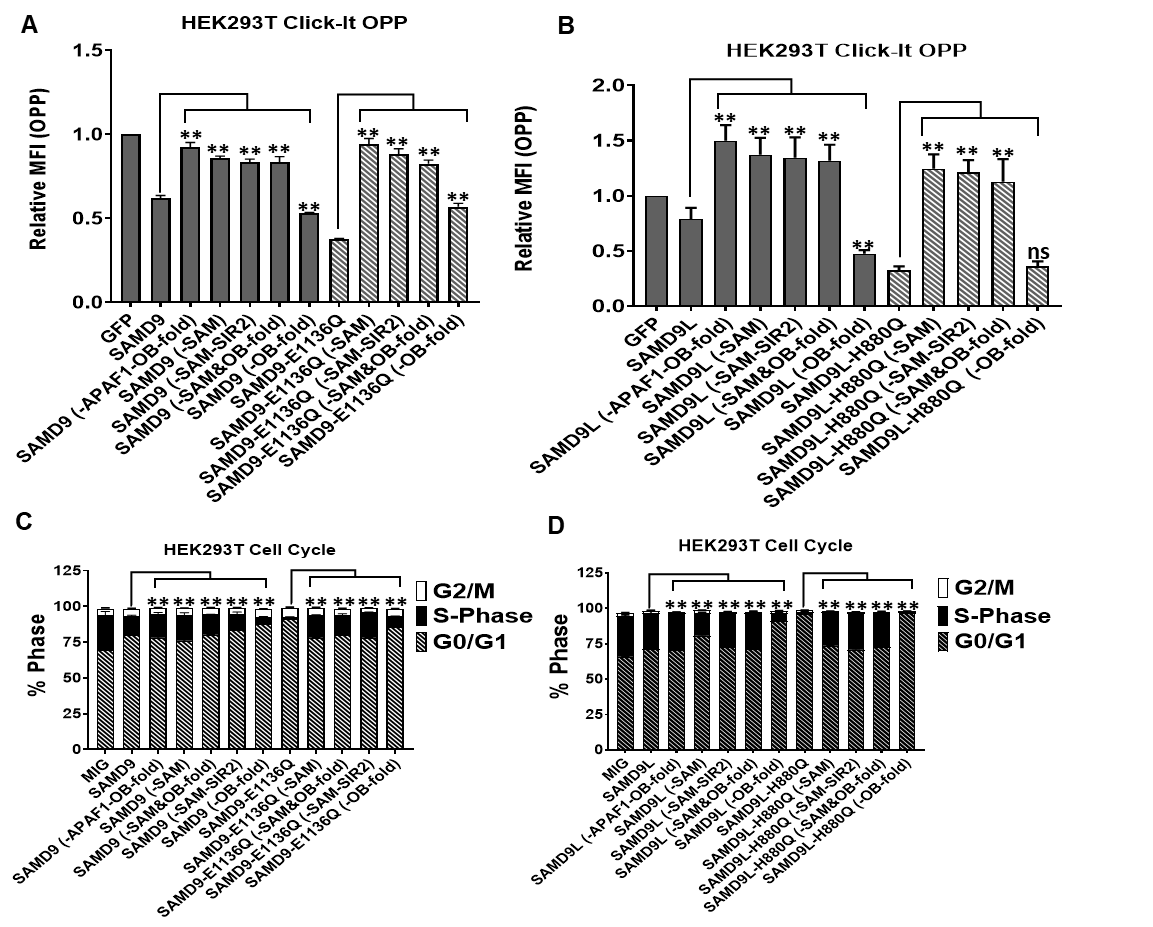


**Figure S5. Functional Analysis of identified functional-domains in SAMD9 and SAMD9L A**-**B**. Flow cytometric analysis of OPP incorporation assay showing relative MFI of HEK293T cells (n=3) after 16hr transfection with (**A**) SAMD9 and (**B**) SAMD9L truncations. **C-D**. Flow cytometric analysis of EdU incorporation in HEK293T cells (n=3) after 16hr transfection with the indicated (**C**) SAMD9 and (**D**) SAMD9L truncations. One-way ANOVA with Bonferroni-correction (ns not significant, * p<0.5, ** p<0.01. Error bars indicate standard error of the mean for three biological replicates compared to relative full-length controls.


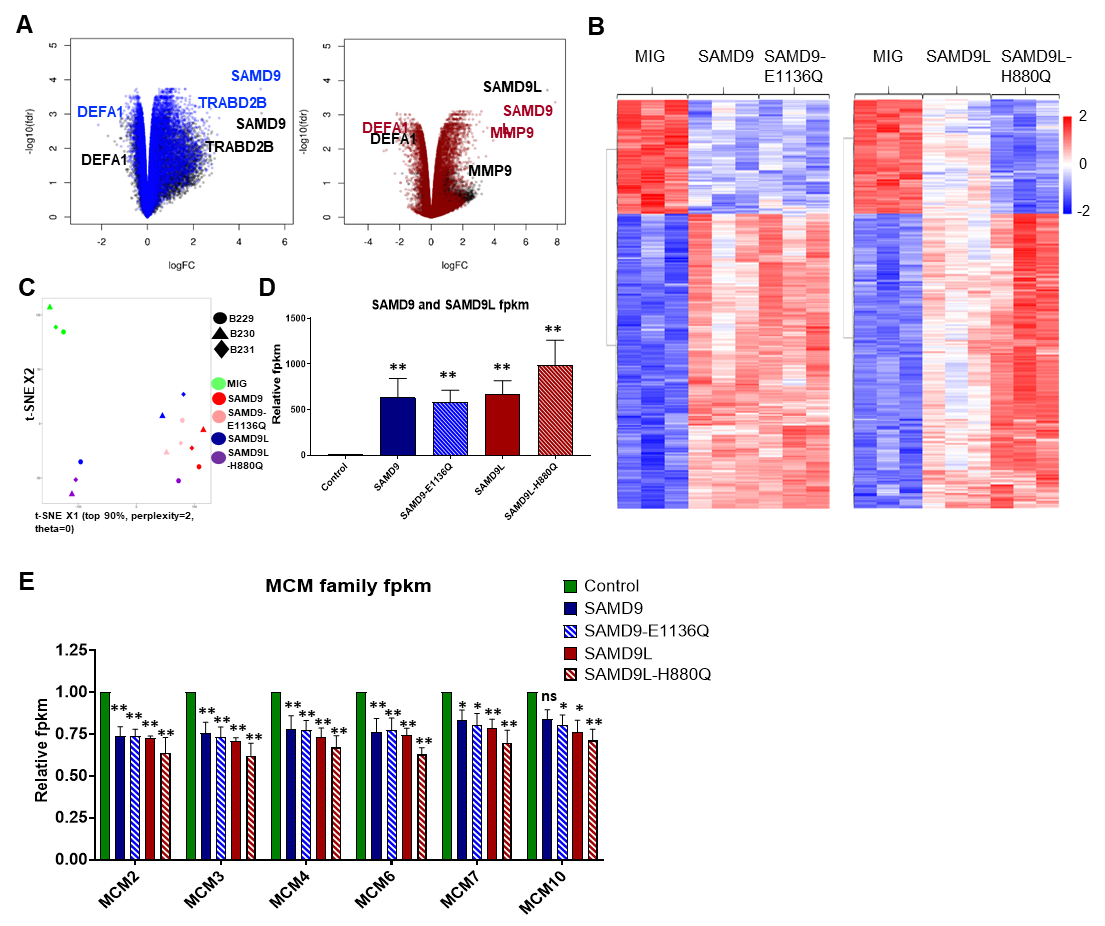


**Figure S6. RNA-seq analysis reveal stress response pathways are regulated by SAMD9 SAMD9L expression. A**. Volcano plots of differentially expressed genes (DEG) for SAMD9-E1136Q in blue overlaid on SAMD9 in black (left) and SAMD9L-H880Q in red overlaid on SAMD9L in black (right). The DEGs were counted and labeled using GENCODE. The plots are labeled with common genes in each group as indicated. **B**. Heatmap of all DEGs (p >0.05) for SAMD9 and SAMD9-E1136Q vs control (left) as well as SAMD9L and SAMD9L-H880Q vs control (right). **C**. tSNE plot demonstrating the clustering of the experimental groups and the CL20 empty vector control. B229, B230, and B231 represent the CD34+ parent lot number each set was derived from. **D**. Relative FPKM (normalized to GFP-vector control) analysis for the overexpression of indicated genes. **E.** Relative FPKM (normalized to GFP-vector control) analysis for the downregulation of the *MCM* family gene expression. One-way ANOVA with Bonferroni-correction (ns not significant, * p<0.5, ** p<0.01. Error bars indicate standard error of the mean for three biological replicates.

**
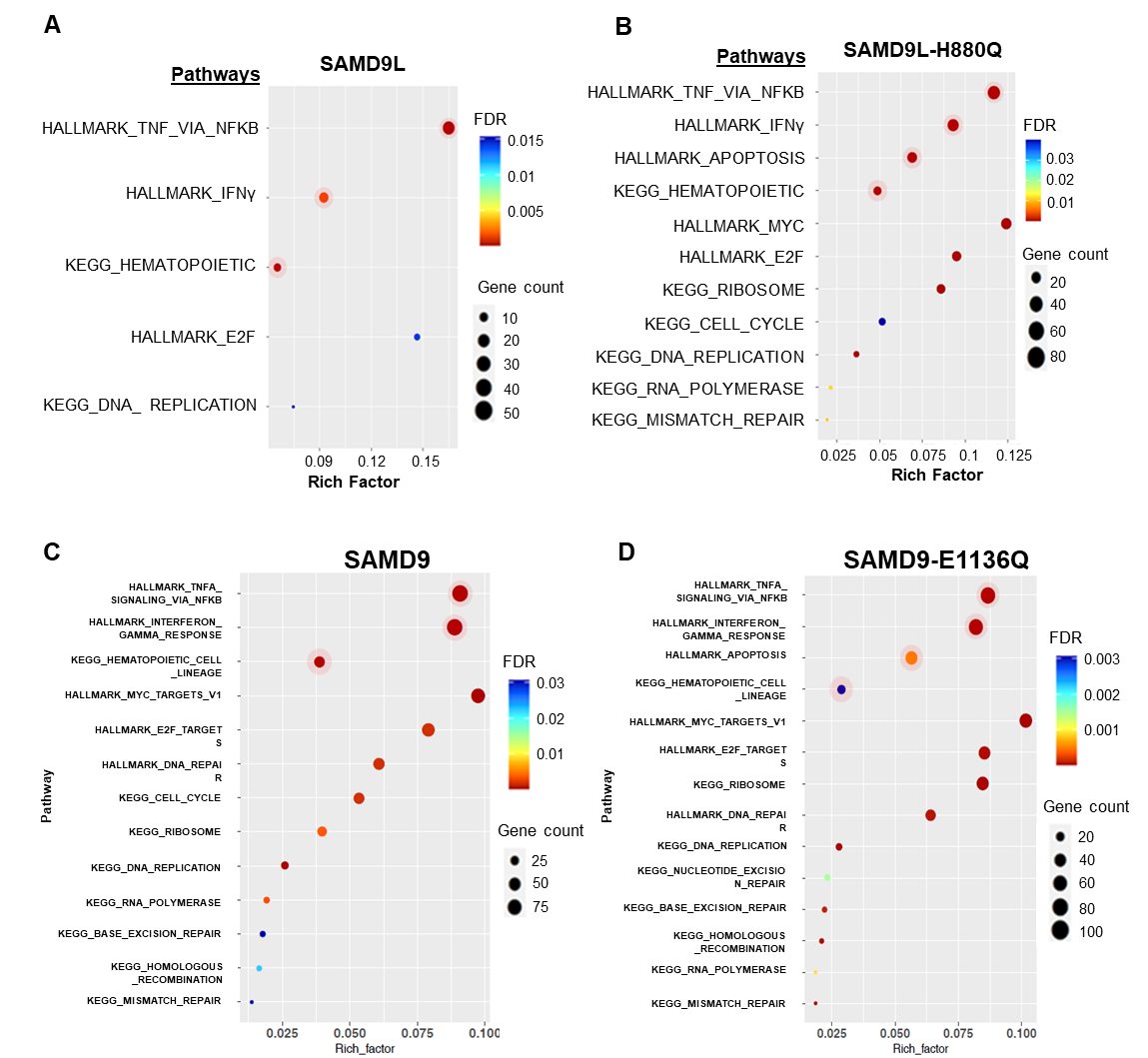
**

**Figure S7. RNA-seq analysis reveals multiple pathways are regulated by SAMD9 and SAMD9L expression. A-D**. Rich factor plots of the DGE pathway enriched in SAMD9L (**A**), SAMD9L-H880Q (**B**), SAMD9 (**C**), and SAMD9-E1136Q (**D**). Rich factor is calculated by statistically significant genes divided by total gene set, size of each dot represents gene count, the color of each dot represents FDR significance for the indicated pathway, and upregulated pathways are indicated with a halo.

**
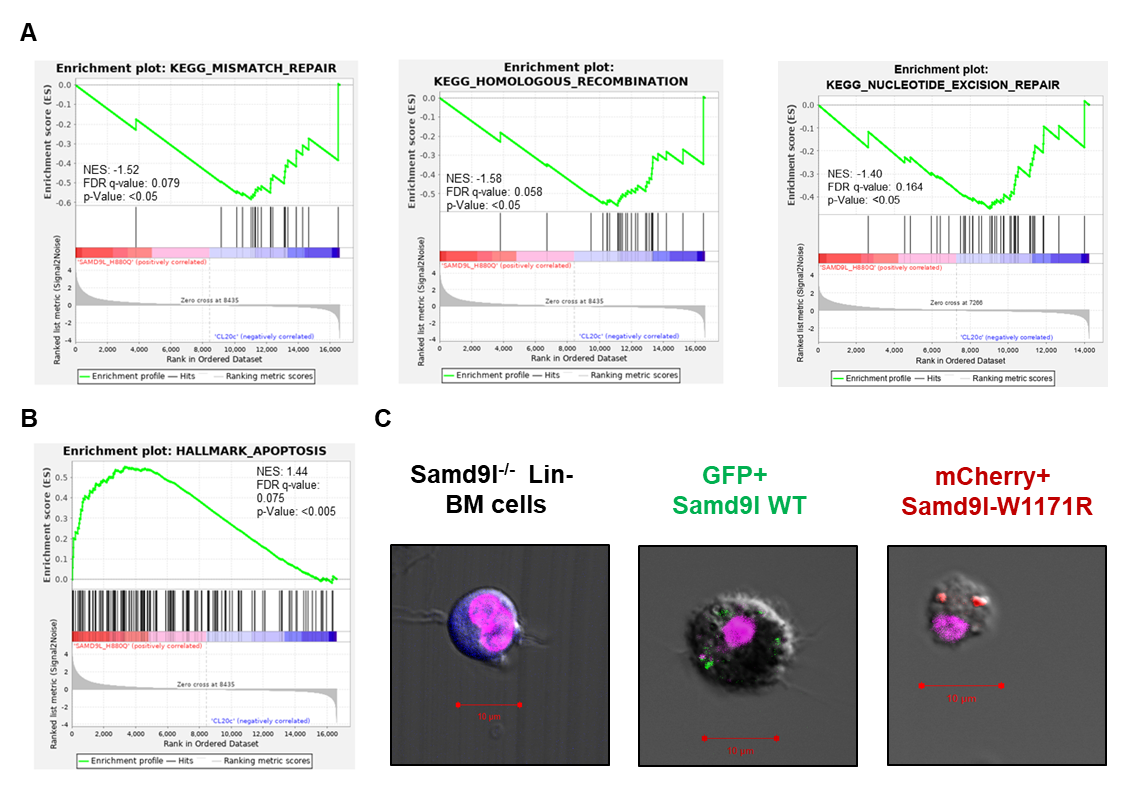
**

**Figure S8. Mutations in SAMD9 and SAMD9L induce DNA damage. A,B**. GSEA illustrating the downregulation of (**A**) DNA repair pathways and upregulation of (**B**) apoptosis in cells expressing SAMD9-H880Q. **C**. Live cell imaging of Samd9l-/- HSPCs transduced with empty-vector or GFP+ Samd9l or mCherry+ Samd9l-W1171R and stained with DRAQ5™ DNA-stain. Images were taken by the Zeiss LSM 780 NLO microscopy using a 60X lens.

**Supplemental Tables**

**Table S1: Table from GSEA of the gene-expression data generated from indicated genes vs. vector control was performed using the Hallmark and KEGG gene sets.**

|  | **SAMD9** | | | **SAMD9-E1136Q** | | | **SAMD9L** | | | **SAMD9L-H880Q** | | |
| --- | --- | --- | --- | --- | --- | --- | --- | --- | --- | --- | --- | --- |
| **GSEA Pathway** | **NES^1^** | **p-value** | **FDR^2^** | **NES^1^** | **p-value** | **FDR^2^** | **NES^1^** | **p-value** | **FDR^2^** | **NES^1^** | **p-value** | **FDR^2^** |
| HALLMARK_MYC_  TARGETS_V2 | -1.5 | 0.00 | 0.064 | -1.7 | 0.00 | 0.028 | -2.3 | 0.00 | 0.000 | -1.8 | 0.00 | 0.012 |
| KEGG_BASE_  EXCISION_REPAIR | -1.3 | 0.04 | 0.149 | -1.4 | 0.00 | 0.074 | -1.3 | 0.09 | 0.119 | -1.0 | 0.44 | 0.738 |
| KEGG_DNA_  REPLICATION | -1.6 | 0.00 | 0.044 | -1.7 | 0.00 | 0.032 | -2.2 | 0.00 | 0.000 | -1.8 | 0.01 | 0.014 |
| KEGG_HOMOLOGOUS_  RECOMBINATION | -1.6 | 0.00 | 0.056 | -1.7 | 0.00 | 0.032 | -1.8 | 0.00 | 0.005 | -1.6 | 0.02 | 0.058 |
| KEGG_MISMATCH_REPAIR | -1.4 | 0.03 | 0.081 | -1.6 | 0.02 | 0.031 | -1.9 | 0.00 | 0.001 | -1.5 | 0.03 | 0.079 |
| KEGG_NUCLEOTIDE_  EXCISION_REPAIR | -1.4 | 0.00 | 0.086 | -1.6 | 0.06 | 0.031 | -2.0 | 0.00 | 0.001 | -1.5 | 0.02 | 0.075 |
| KEGG_RIBOSOME | -1.1 | 0.00 | 0.366 | -1.8 | 0.00 | 0.008 | n/a | n/a | 1.000 | -2.1 | 0.00 | 0.000 |
| KEGG_RNA_POLYMERASE | -1.5 | 0.06 | 0.079 | -1.5 | 0.03 | 0.041 | -1.8 | 0.00 | 0.007 | -1.7 | 0.00 | 0.025 |
| HALLMARK_TNFA_  SIGNALIN_VIA_NFKB | 1.1 | 0.25 | 0.667 | 1.1 | 0.06 | 0.479 | 1.2 | 0.02 | 0.331 | 1.8 | 0.00 | 0.003 |
| HALLMARK_APOPTOSIS | 0.9 | 0.77 | 0.930 | 1.0 | 0.42 | 0.700 | 1.0 | 0.53 | 0.861 | 1.4 | 0.00 | 0.075 |
| HALLMARK_INFLAMMATORY_ RESPONSE | 1.2 | 0.03 | 0.365 | 1.1 | 0.03 | 0.401 | 1.2 | 0.00 | 0.337 | 1.7 | 0.00 | 0.004 |
| KEGG_HEMATOPOIETIC_  CELL_LINEAGE | 1.1 | 0.11 | 0.369 | 1.2 | 0.08 | 0.380 | 1.2 | 0.03 | 0.332 | 1.7 | 0.00 | 0.004 |
| NES: Normalized enrichment score, FDR: False Discovery Rate | | | | | | | | | | | | |

**Table S2: Primers and antibodies**

| **Gene** | **Domain(s) deleted** | **amino acid number** | **Sequence (5`-3`)** |
| --- | --- | --- | --- |
| **SAMD9** | SAM | 166-end | F-CACCATGTTTGATGAATTCAGTAATCCATATCGTTACAAG |
|  |  |  | R-TTACTTGTCGTCATCGTCTTTGTAGTCCATAACAATTTCAATGTCATAAG |
|  | Apaf1-like, OB-fold | 1-579 | F-CACCATGGCAAAGCAACTTAACCTTCC |
|  |  |  | R-TTACTTGTCGTCATCGTCTTTGTAGTCCATGTGCACACAAATACACAGTATATTTTCCATTCC |
|  | SAM, Alba, Sir-2 | 568-end | F-CACCATGAAAGGAATGGAAAATATACTGTGTATTTGTGTG |
|  |  |  | R-TTACTTGTCGTCATCGTCTTTGTAGTCCATAACAATTTCAATGTCATAAG |
|  | SAM,  OB-fold | 185-1179 | F-CACCATGACAGGACCAGGCAATCTCATTGATCCGATACATG |
|  |  |  | R-TTACTTGTCGTCATCGTCTTTGTAGTCCATTCTTTCCTTCACTTCATACTCTCTATCTTCACT |
|  | OB-Fold | 1-1179 | F-CACCATGGCAAAGCAACTTAACCTTCC |
|  |  |  | R-TTACTTGTCGTCATCGTCTTTGTAGTCCATTCTTTCCTTCACTTCATACTCTCTATCTTCACT |
| **SAMD9L** | SAM | 173-end | F-CACCATG GACAGCCATCGCTACATAGAACATTATACTCTA |
|  |  |  | R-TTACTTGTCGTCATCGTCTTTGTAGTCCATAATTACTTCTATATCATATGCCAGA |
|  | Apaf1-like, OB-fold | 1-583 | F-CACCATGAGTAAACAAGTATCTCTACC |
|  |  |  | R-TTACTTGTCGTCATCGTCTTTGTAGTCCATGTTTACAGAGATACACAACATATTTTC |
|  | SAM, Alba, Sir-2 | 573-end | F-CACCATG AAAGGAATGGAAAATATGTTGTGTATCTCTGTA |
|  |  |  | R-TTACTTGTCGTCATCGTCTTTGTAGTCCATAATTACTTCTATATCATATGCCAGA |
|  | SAM, OB-fold | 187-1179 | F-CACCATG ACAGGAGCACTCAATCTCATTGATCCAATACAT |
|  |  |  | R-TTACTTGTCGTCATCGTCTTTGTAGTCCATGTTCTCGGTTTCATAGTTTTTACT |
|  | OB-Fold | 1-1179 | F-CACCATGAGTAAACAAGTATCTCTACC |
|  |  |  | R-TTACTTGTCGTCATCGTCTTTGTAGTCCATGTTCTCGGTTTCATAGTTTTTACT |
| **Antibody** | **Conjugate/ Fluorophore** | **Company** |  |
| SAMD9 | n/a | abcam 180575 | |
| SAMD9L | n/a | Proteintech 25173-1 | |
| GFP | n/a | ThermoFisher A-11122 | |
| DHX9 | n/a | Bethyl Laboratories A300-855 | |
| DNA-PKcs | n/a | Bethyl Laboratories A300-516 | |
| DDX1 | n/a | Bethyl Laboratories A300-521 | |
| eIF3A | n/a | Bethyl Laboratories A302-003 | |
| RPS6 | n/a | Bethyl Laboratories A300-557A | |
| RPL11 | n/a | Bethyl Laboratories A303-931A | |
| αTubulin | n/a | Bethyl Laboratories A305-798A | |
| SF3B1 | n/a | MBL Life Science D138-3 | |
| Rab5 | n/a | BD Bioscience 610724 | |
| Donkey anti-Rabbit | AlexaFluor 568 | ThermoFisher A10042 | |
| Goat anti-Mouse | AlexaFluor 647 | ThermoFisher A21235 | |
| γH2AX | AlexaFluor 647 | Biolegend 613416 | |
| Annexin-V | AlexaFluor 647 | BD Bioscience 550474 | |

***Table S3: CRISPR construct sequences and screening primers.***

| **Name** | **Sequence (5’ to 3’)** |
| --- | --- |
| hSAMD9 sgRNA spacer (SS11.SAMD9.g5) | UGGUGGAUAGAGGAAAACGG |
| hSAMD9_E1136Q_ssODN  (SS11.hSAMD9.g5.block.sense)  E1136Q mutation and blocking modifications to prevent sgRNA recutting subsequent to integration are in upper case.  *phosphorothioate linkages | t*t*atatctcagatacactgggtcaagtctacaaaagtaaaataagatggtggataGAGgaGaaTggaggaaacgggaacatttcagttgatgatctaattgctcttttggatttagcagaac*a*t |
| SS11.hSAMD9.DS.F | CCAAGCGTTGGCAAGACATTTCTACA |
| SS11.hSAMD9.DS.F | GCTGGAGAATTTGGATTGTGTAAAGCCC |

**Supplemental References**

1. Sentmanat MF, Peters ST, Florian CP, Connelly JP, Pruett-Miller SM. A Survey of Validation Strategies for CRISPR-Cas9 Editing. Sci Rep. 2018;8(1):888.

2. Connelly JP, Pruett-Miller SM. CRIS.py: A Versatile and High-throughput Analysis Program for CRISPR-based Genome Editing. Sci Rep. 2019;9(1):4194.

3. Schwartz JR, Ma J, Lamprecht T, Walsh M, Wang S, Bryant V, et al. The genomic landscape of pediatric myelodysplastic syndromes. Nat Commun. 2017;8(1):1557.

4. Drozdetskiy A, Cole C, Procter J, Barton GJ. JPred4: a protein secondary structure prediction server. Nucleic Acids Res. 2015;43(W1):W389-94.

5. Mekhedov SL, Makarova KS, Koonin EV. The complex domain architecture of SAMD9 family proteins, predicted STAND-like NTPases, suggests new links to inflammation and apoptosis. Biol Direct. 2017;12(1):13.

6. Xu P, Duong DM, Peng J. Systematical optimization of reverse-phase chromatography for shotgun proteomics. J Proteome Res. 2009;8(8):3944-50.

7. Washburn MP. The H-index of 'an approach to correlate tandem mass spectral data of peptides with amino acid sequences in a protein database'. J Am Soc Mass Spectrom. 2015;26(11):1799-803.

8. Zhou JY, Afjehi-Sadat L, Asress S, Duong DM, Cudkowicz M, Glass JD, et al. Galectin-3 is a candidate biomarker for amyotrophic lateral sclerosis: discovery by a proteomics approach. J Proteome Res. 2010;9(10):5133-41.

9. Chu Q, Rathore A, Diedrich JK, Donaldson CJ, Yates JR, 3rd, Saghatelian A. Identification of Microprotein-Protein Interactions via APEX Tagging. Biochemistry. 2017;56(26):3299-306.

10. Panda AC, Martindale JL, Gorospe M. Polysome Fractionation to Analyze mRNA Distribution Profiles. Bio Protoc. 2017;7(3).

11. Faber ZJ, Chen X, Gedman AL, Boggs K, Cheng J, Ma J, et al. The genomic landscape of core-binding factor acute myeloid leukemias. Nat Genet. 2016;48(12):1551-6.

12. Z. Y. and T. L. Leek JT JW, Parker HS, Fertig EJ, Jaffe AE, Storey JD. sva: Surrogate Variable Analysis. R package version 3.26.0. 2017.

13. Ritchie ME, Phipson B, Wu D, Hu Y, Law CW, Shi W, et al. limma powers differential expression analyses for RNA-sequencing and microarray studies. Nucleic Acids Res. 2015;43(7):e47.

14. Yu G, Wang LG, Han Y, He QY. clusterProfiler: an R package for comparing biological themes among gene clusters. Omics. 2012;16(5):284-7.

15. Subramanian A, Tamayo P, Mootha VK, Mukherjee S, Ebert BL, Gillette MA, et al. Gene set enrichment analysis: a knowledge-based approach for interpreting genome-wide expression profiles. Proc Natl Acad Sci U S A. 2005;102(43):15545-50.
